# Anticancer Effect of *Salvia plebeia* and Its Active Compound by Improving T-Cell Activity *via* Blockade of PD-1/PD-L1 Interaction in Humanized PD-1 Mouse Model

**DOI:** 10.3389/fimmu.2020.598556

**Published:** 2020-11-05

**Authors:** Jang-Gi Choi, Young Soo Kim, Ji Hye Kim, Tae In Kim, Wei Li, Tae Woo Oh, Chang Hyun Jeon, Su Jin Kim, Hwan-Suck Chung

**Affiliations:** Korean Medicine (KM) Application Center, Korea Institute of Oriental Medicine, Daegu, South Korea

**Keywords:** PD-1, PD-L1, immune checkpoint, humanized PD-1 mouse, traditional oriental medicine, *Salvia plebeia* R. Br., cosmosiin

## Abstract

Immune checkpoint inhibitors, increasingly used to treat malignant tumors, are revolutionizing cancer treatment by improving the patient survival expectations. Despite the high antitumor efficacy of antibody therapeutics that bind to PD-1/PD-L1, study on small molecule-based PD-1/PD-L1 inhibitors is required to overcome the side effects of antibody therapeutics caused by their size and affinity. Herein, we investigated antitumor potential of *Salvia plebeia* R. Br. extract (SPE), which has been used as a traditional oriental medicine and food in many countries, and its components by the blockade of PD-1/PD-L1 interaction. SPE and its component cosmosiin effectively blocked the molecular interaction between PD-1 and PD-L1. SPE also inhibited tumor growth by increasing CD8+ T-cells in the tumor through the activation of tumor-specific T-cells in a humanized PD-1 mouse model bearing hPD-L1 knock-in MC38 colon adenocarcinoma tumor. This finding presents a preclinical strategy to develop small molecule-based anticancer drugs targeting the PD-1/PD-L1 immune checkpoint pathway.

## Introduction

Immune checkpoint inhibitors are being increasingly used in the treatment of malignant tumors, and they are revolutionizing treatment approaches and increasing survival expectations in cancer patients (1, 2). The programmed cell death (PD-1) pathway is an attractive therapeutic target in many cancers. Previous studies have reported that blocking of the interaction of PD-1 with its ligands PD-L1 (programmed cell death ligand 1) and PD-L2 can result in remarkable antitumor responses and clinical benefits in some patients (3, 4). PD-L1 is expressed in various types of tumors, including melanoma and stomach, breast, and lung cancers, as well as immune cells that penetrate the tumor in the tumor microenvironment (5). Blocking of the PD-1/PD-L1 interaction appears to allow cytotoxic T-cells to attack cancer cells (3). Thus, various efforts are being made to develop PD-1/PD-L1 inhibitors for the treatment of human cancers (2, 3, 6). To date, the FDA has approved six drugs (pembrolizumab, nivolumab, atezolizumab, avelumab, durvalumab, and cemiplimab) that target the PD-1/PD-L1 pathway for cancer immunotherapy, and many studies are currently underway to obtain additional approvals (7). However, antibody therapies have issues, such as immune-related adverse events (irAEs), inadequate pharmacokinetics and tumor penetration, and high costs for manufacturers and consumers (8). In particular, antibody therapies have sometimes been reported to cause serious grade 3 and 4 adverse events; up to 55% of ipilimumab-treated patients, up to 43% of nivolumab-treated patients, 11–14% of pembrolizumab-treated patients, and 54–86% of ipilimumab plus nivolumab-treated patients (9). These issues have increased interest in the development of alternative small molecule interventions that include peptidomimetics and peptides targeting the PD-1/PD-L1 immune checkpoint pathway and interfere with the broad clinical application of antibodies (10, 11). Although small molecule-based immune checkpoint inhibitors may be digested rapidly in the human body compared to antibody drugs, their rapid clearance makes it possible to reduce irAEs by providing flexibility to adjust drug dose and medication schedule (12). Small molecules for targeting the PD-1/PD-L1 immune checkpoint pathway are under development, but no molecule has yet been approved (11).

The development of anticancer drugs derived from medicinal herbs has played an important role in the effective treatment of cancer patients (13, 14). In addition, medicinal herbs have long been used as anticancer materials and folk remedies by extracting active ingredients and developing them into drugs (13, 14). In order to overcome the occurrence of anticancer drug resistance, various biological resources, including medicinal herbs, have been assessed for the development of anticancer drugs, and their importance is increasing (13–15).

*Salvia plebeia* R. Br. (SP) is an edible plant widely distributed in many countries, including Korea, India, and China and has been used as a folk medicine to treat common cold, diarrhea, and hepatitis (16, 17). SP mainly contains diverse phytochemical substances, such as flavonoids (18), sesquiterpenoids (19), monoterpene glycosides, steroids, and different types of phenolic compounds (17). Recent studies have reported that SP ethanol extract (SPE) has a wide range of biological activities, such as anti-inflammatory (20), antiviral (17, 21), antioxidant (22), antiangiogenic, and antinociceptive activities (23). However, to our knowledge, there is no report on the antitumor activity of SPE and its active compounds targeting of the PD-1/PD-L1 immune checkpoint pathway.

The present study investigated the ability of SPE and its components to block the PD-1/PD-L1 interaction, using various *in vitro* biochemical and cell-based assays.

Efforts have been undertaken for the development of therapeutic antibodies targeting human PD-1/PD-L1 (hPD-1/hPD-L1) to treat various types of human cancer. However, to date, no animal model is suitable for evaluating the antitumor efficacy of such antibodies targeting hPD-1/hPD-L1 (24). Therefore, in present study, for investigating the anti-tumor effect of SPE, we used hPD-L1 knock-in MC38 tumor-bearing humanized PD-1 mouse model to study successfully established tumor immunotherapies.

The findings of this study could help in the development of PD-1 and PD-L1 inhibitors and could support the potential use of SPE in tumor immunotherapies.

## Materials and Methods

### SP Preparation

SP was purchased from the National Development Institute of Korean Medicine (Gyeongsan, South Korea). Freeze-dried extract powder was dissolved in 50% DMSO and centrifuged at 12,000 rpm for 30 min to remove insoluble residues. The supernatant was stored in desiccators at 4°C until further use.

### Reagents

Caffeic acid (#CFN99190), luteolin (#CFN98784), luteolin 7-O-glucoside (#CFN98565), cosmosiin (#CFN98981), homoplantaginin (#CFN90344), apigenin (#CFN98843), and hispidulin (#CFN99491) were purchased from ChemFaces (Wuhan, China). PD-1/PD-L1 inhibitor screening assay kit (#72005), CTLA-4 (cytotoxic T-lymphocyte-associated protein 4)/CD80 inhibitor screening assay kit (#72009), and PD-L1-blocking antibody (#71213) were purchased from BPS Bioscience (San Diego, CA, USA). Small molecule PD-L1 inhibitor C1 (BMS-1, # T3655) was purchased from TargetMol (Wellesley Hills, MA, USA). Anti-PD-1 antibody (Pembrolizumab, #A2005) and anti-CTLA-4 antibody (#A2001) was purchased from Selleck Chemicals (Houston, TX, USA).

### Cell Lines

An immortalized human T lymphocyte Jurkat cells (ATCC TIB-152) cells were obtained from American Type Culture Collection (Manassas,VA, USA). Cells were maintained in RPMI 1640 medium containing 10% fetal bovine serum (FBS) and 1% penicillin and at 37°C in a 5% CO_2_ incubator. Jurkat cells were infected with control or PD-1-expressing lentiviruses (GeneChem, Shanghai). hPD-L1 and TCR agonist (aAPC/CHO-K1) cells were infected with control or PD-L1-expressing lentiviruses (GeneChem, Shanghai). Cell surface PD-1 or PD-L1 expression was confirmed by FACS analysis in infected cells (PD-1 Jurkat or PD-L1 aAPC/Cho-K1 cells). Humanized PD-L1 in MC38 cells (hPD-L1-MCs) derived from C57BL/6 murine colon adenocarcinoma cells were purchased from Shanghai Model Organisms Center (Shanghai, China). hPD-L1-MCs were maintained in Dulbecco’s modified Eagle medium (DMEM) containing 10% and 1% penicillin and streptomycin at 37°C in a 5% CO_2_ incubator. All cell culture solutions were purchased from Hyclone Laboratories Inc. (GE Healthcare Life Sciences, Chicago, IL, USA).

### Competitive ELISA

Competitive ELISA was performed using a PD-1/PD-L1 or CTLA-4/CD80 inhibitor ELISA-screening kit (BPS Bioscience), according to the manufacturer’s instructions (25). Briefly, recombinant hPD-L1 (#71104, BPS Bioscience) or CTLA-4 (#71149, BPS Bioscience) was coated overnight at 1 μg/mL in PBS onto 96-well plates (Corning Inc., New York, NY, USA). The plates were washed with PBS containing 0.1% tween (PBS-T), blocked for 1 h at room temperature with PBS containing 2% (w/v) BSA, and washed again. Then, 5 µL of 0.5 μg/mL biotinylated hPD-1 (#71109, BPS Bioscience) or biotinylated hCD80 (#71114, BPS Bioscience) was added to the wells, and the plates were incubated for 2 h at room temperature. After three washes in PBS-T, 50 μL of 0.2 μg/mL HRP-conjugated streptavidin was added to each well, and the plates were incubated for 1 h. After incubation, plates were washed thrice with 0.1% PBS-T, and relative chemiluminescence was measured using a SpectraMax L Luminometer (Molecular Devices, San Jose, CA, USA).

### PD-1/PD-L1 Blockade Bioassay

To confirm the efficacy of SPE and its components for inhibiting the PD-1/PD-L1 interaction, a cell-based PD-1/PD-L1 blockade bioassay was performed using PD-1/PD-L1 blockade bioassay (#J4011, Promega, Madison, WI, USA) with slight modification of the manufacturer’s instructions. In brief, 5 × 10^4^ PD-L1 aAPC/CHO-K1 cells were seeded into 96-well plates in DMEM with 10% FBS. On the day of the assay, the medium was aspirated, and then, PD-L1-blocking antibody or SPE/components, as indicated, and 1 × 10^5^ PD-1 effector cells were added. Following mixing with Bio-Glo™ Reagent (Promega, # G7940), luminescence was measured using the GloMax^®^ Explorer Multimode Microplate Reader (Promega). The data of the PD-1/PD-L1 blockade bioassay are presented as mean ± standard error of the mean of four independent experiments.

### Cell Counting Kit-8 (CCK) Assay

Cell viability was measured using CCK (#CK04, Dojindo Molecular Technologies, Inc., Rockville, MD, USA), according to the manufacturer’s instructions. Briefly, cells were seeded into 96-well plates at a density of 1 × 10^4^ cells/well and were cultured overnight before SPE and cosmosiin treatment. SPE (0–400 µg/mL) and cosmosiin (0–5 µM) were added to the wells at various concentrations (0–400 µg/mL). After incubation for the indicated time, 10 μL of CCK solution was added, and the mixture was kept for 2 h at 37°C. A microplate reader (Molecular Devices i3, San Jose, CA, USA) was used to measure color intensity at 450 nm.

### Murine Splenocytes or Tumor Infiltrating T cells (TILs) Isolation

A humanized PD-1 mouse model (genetic background of C57BL/6J; genetically modified for expression of the human full-length PD-1 protein) was purchased from Shanghai Model Organisms Center (Shanghai, China). The animal study was carried out in accordance with the guidelines of the Institutional Animal Care and Use Committee (IACUC) of the Korea Institute of Oriental Medicine (KIOM), and were approved by the IACUC of the KIOM (approval number: KIOM-D-19-003). Humanized PD-1 mouse splenocytes (hPD1-MSs) were isolated as previously explained (26). For extraction of TILs (CD8+ T cells), tissue from MC38 tumor tissues were cut and digested with collagenase (0.5 mg/ml collagenase IV) at 37 °C for 1* h*. Cell suspension was then twice filtered through 100-μm and 40-μm cell strainers (SPL) to obtain single cells. TILs were isolated from single cells were conducted by negative selection beads as per the manufacturer*’*s protocols (STEMCELL Technologies Inc., Vancouver, Canada).

### Co-Culture Experiments and In Vitro T cell Killing Assay

The hPD-L1-MCs were stained using the CellTrace™ Far Red Cell Proliferation Kit (ThermoFisher Scientific, Waltham, MA, USA), according to the manufacturer*’*s protocol. The hPD1-MSs or CD8+ T-cells were used as effector cells and hPDL1-MCs were used as target cells. Effector cells were activated with Dynabeads Mouse T-Activator CD3/CD28 (Life Technologies, Carlsbad, CA, USA) for 3 days in a 6-well plate. After activation, hPD1-MSs were added to 200 µL of culture medium in the wells of a 96-well culture plate containing 1 × 10^4^ CellTrace™ Far Red-labeled hPDL1-MCs at an effector cell-to-target cell ratioof 5:1. After incubation for 72* h*, cocultured cells were observed under a fluorescence microscope (Olympus, Tokyo, Japan) and cytotoxicity assay were performed as descibed above. Human PD-L1 expression in murine splenocytes from humanized PD-1 mice were analyzed by flow cytometry with slight modification of a previous study (24).

### High Performance Liquid Chromatography (HPLC) Analysis

The four SPE components cosmosiin, homoplantaginin, apigenin, and hispidulin were found to be involved in PD-1/PD-L1 interaction blockade. The amounts of these four components were analyzed using previously reported methods with slight modification (27). We analyzed the HPLC profiles of SPE (4 mg/mL) and SPE fractions (MC, 4 mg/mL; EA, 1 mg/mL; BuOH, 1 mg/mL) using Alliance e2695 (Waters Corp., Milford, MA, USA) by injecting 10 μL of sample into a Geminin C18 column (5 μm, 250 × 4.6* mm*; Phenomenex Inc., Torrance, CA, USA) at an oven temperature of 40°C. The mobile phase was applied at a flow rate of 1.0 mL/min with a gradient of acetonitrile (A) and distilled water (B) containing 1% acetic acid as follows: 15% A (0–2 min), 15–60% A (2–82 min), 60–100% A (82–83 min), 100% A (83–84 min), 100–15% A (84–85 min), and 15% A (85–90 min). The samples were monitored under UV light at 265 nm. Standard cosmosiin, homoplantaginin, apigenin, and hispidulin were used for reference.

### Kinetic Analysis by Biolayer Interferometry

The binding affinities and kinetic constants of cosmosiin for PD-1 and PD-L1 were measured by biolayer interferometry on a BLItz system (Pall FortéBio Corp., Menlo Park, CA). After the pre-equlibration of the SA BLI sensor (Pall FortéBio Corp., Menlo Park, CA) in PBS buffer for 10* min*, biotinylated hPD-1 (BioVision, Milpitas, CA, USA) and PD-L1 (Sinobiological, Beijing, China) were fully loaded onto the sensors by immersing in 4 μL of PD-1 and PD-L1 solution, dissolved in PBS buffer to 50 μg/mL. Cosmosiin solution for the kinetic analysis was prepared by 100-fold dilution of stock solution, dissolved in 100% DMSO (0, 12.5, 25, and 37.5 mM), in PBS buffer. Binding kinetics were measured as follows: step 1, initial baseline in PBS buffer containing 1% DMSO for 15 s; step 2, association in 4 μL of cosmosiin solution for 20 s; and step 3, dissociation in PBS buffer containing 1% DMSO for 20 s. The kinetic constants were calculated using the BLItz Pro software by fitting the association and dissociation data to a 1:1 model. The equilibrium dissociation constant, K_D_, was calculated as dissociation constant (k_d_)/association constant (k_a_).

### Docking Simulation and Interaction Analysis

The SPE component cosmosiin was docked onto the interaction space between PD-1 and PD-L1 retrieved from the Protein Data Bank (www.rcsb.org, PDB code: 4ZQK) and a previous report (28), using AutoDock Vina integrated with UCSF Chimera-alpha v1.13 (29). The binding affinity between PD-L1/PD-1 and small molecule cosmosiin was expressed as the lowest energy score in the binding simulation. The hydrophobic and hydrogen bonding interactions between PD-L1 and each small molecule were analyzed using LigPlot+ v1.4.5 (30). Amino acid residues involved in the interactions were indicated with red (hydrophobic interactions) and green (H-bonds).

### Tumor Cell Inoculation and In Vivo Analysis

A humanized PD-1 mouse model (genetic background of C57BL/6J; genetically modified for expressing the human full-length PD-1 protein) was purchased from Shanghai Model Organisms Center (Shanghai, China). Experiments were performed in accordance with the guidelines of the Institutional Animal Care and Use Committee (IACUC) of the Korea Institute of Oriental Medicine (KIOM), and were approved by the IACUC of the KIOM (approval number: KIOM-D-19-003). All mice were maintained in a SPF facility at KIOM. All annimals (n=6) were exposed to 2% isoflurane mixed with oxygen for anesthesia using Avesko inhalation anesthesia system (31, 32). To establish subcutaneous tumor, humanized PD-1 mice were inoculated with hPD-L1 MC38 cells in the right flank with 200 μL/mouse of the cells adjusted to 3.0 × 10^6^ cells/mL (1.5 × 10^5^ cells/mouse). Tumor growth was monitored and measured twice a week using digital caliper (Hi-Tech Diamond, Westmont, IL, U.S.), and the tumor volume was calculated according to the following formula: (W^2^ × L)/2 (W: short diameter; L: long diameter). When the tumor volume reached 100 mm^3^ (day 14), mice were randomized into groups of 6 animals per group: an untreated control group (no tumor cells injected-group), and groups injected with MC38 tumor cells plus vehicle (PBS, 10 mL/kg, q.d., i.g.), Anti-PD-1 antibody with pembrolizumab (5 mg/kg, b.i.w.x2, i.p.), a low or high dose of SPE (100 or 300 mg/kg, q.d., i.g.). On days 1, 4, 8, 11, and 15 post implantation, mice were dosed intraperitoneally (i.p.) with PBS or anti-PD-1 antibody. Intragastric (i.g.) injection were daily given with SPE for 15 days. All mice were sacrificed for analyses on 16 day after the treatment.

### Tissue Dissociation for Cell Isolation and Flow Cytometry

Tumor tissues were harvested in RPMI Medium 1640 with 10% heat-inactivated FBS, 1% penicillin and streptomycin (all from HyClone™). Tumors were minced and incubated for 30 min in DPBS (Well-gene) with 0.5 mg/ml Collagenase IV (Sigma) at 37 °C. Tissues were squeezed through a 100 μm cell strainer (# 93100, SPL), re-filtered through a 70 μm cell strainer (# 93070, SPL) subjected for 5 min to Red Blood Cell Lysis Buffer (#10-548E, Lonza). Flow cytometry was performed to determine cell surface antigen expression by 30-min incubation on ice with pertinent antibodies. The following monoclonal antibodies were used: mouse-specific monoclonal antibodies used were PerCP/Cyanine5.5 anti-mouse CD45 (#103131, BioLegend), APC-anti-mouse CD8a (#100712, BioLegend) and corresponding isotype control mAbs PerCP/Cy5.5 Rat IgG2b (#400632, BioLegend) and APC Rat IgG2a (#400512, BioLegend). The cells were washed thrice with PBS containing 2% FBS and fixed in suspension with 4% paraformaldehyde and stored at 4 °C until analysis with a CytoFLEX flow cell counter (Beckman). We analyzed the data using FlowJo software.

### Staining for Immunofluorescence

After tissue fixation, dehydrated and embedded in paraffin before sections by cryo ultramicrotome (Leica, Wetzlar, Germany) into serial 10-μm sections. All histological sections were divided two parts. One part was stained with hematoxylin-eosin (H&E) staining photographed to determine cancer saturation rate and normal cells were confirmed and morphological changes. The other part was subjected to immunochemistry staining using. The sections were deparaffinized and non-specific endogenous peroxidase activity blocked with 3.0% H2O2 for 10-min at room temperature (RT). After washing with TBST, the sections were reacted with Anti-CD3 mAb (1:50, ab16669, Abcam, Cambridge, MA, USA) as a T-cell activation marker for 24* h* at 4°C, after being incubated with the secondary antibody (Rabbit/Mouse, Dako REAL™ EnVision™ Detection System, K500711) for 30* min* at room temperature, specimens were stained with DAB (3, 3-diaminobenzidine; Dako REAL™ EnVision™ Detection System, Peroxidase/DAB+, K500711). The sections were counterstained with hematoxylin, dehydrated and embedded with permount. Histopathological changes of ischemic brains were observed under microscope with 400× magnification.

### Statistical Analysis

Data are presented as mean ± standard error of the mean. Statistical significance was determined using one-way ANOVA with Tukey’s post-hoc test for multiple comparisons, with consideration of the mean difference between the treatment and control groups. All statistical analyses were performed using GraphPad PRISM software (v5.02; La Jolla, CA, USA). A *p*-value <0.05 was considered to indicate statistical significance.

## Results

### PD-1/PD-L1 Interaction Blockade With SPE

**Figure 1:**
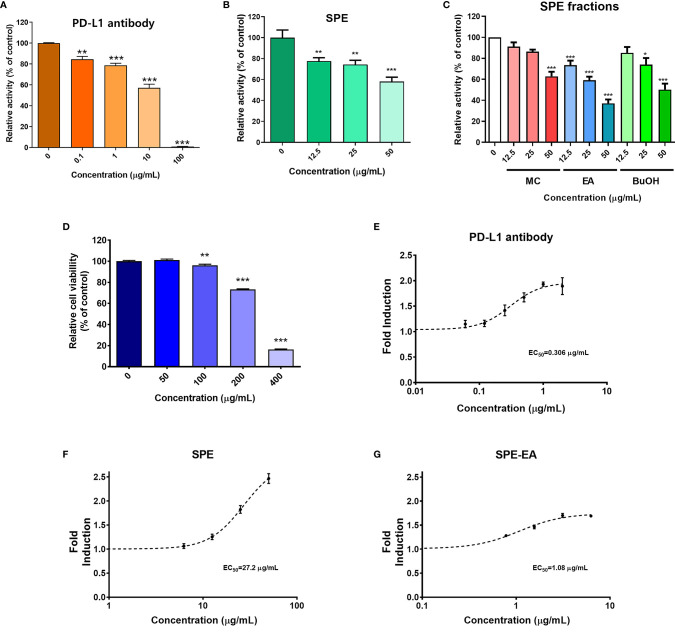
PD-1/PD-L1 interaction blockade by *Salvia plebeia* R. Br. ethanol extract (SPE). Competitive ELISA was performed using PD-1/PD-L1 inhibitor ELISA-screening assay with the indicated concentrations of PD-L1-blocking antibody, SPE, and SPE fractions. **(A)** Inhibition of the PD-1/PD-L1 interaction by PD-L1-blocking antibody, **(B)** SPE, and **(C)** SPE fractions (methylene chloride [MC], ethyl acetate [EA], and n-butanol [BuOH]). Cytotoxicity and T-cell activation by SPE and its ethyl acetate (EA) fraction *in vitro*. **(D)** The viabilities of PD-L1 aAPC/CHO-K1 and PD-1 effector cells were assessed using the Cell Counting Kit-8 (CCK) assay after treatment with the indicated concentrations of SPE for 24 h. **(E–G)** A PD-1/PD-L1 blockade bioassay was performed to assess the inhibitory activities of PD-L1-blocking antibody, SPE, and SPE-EA. PD-L1 aAPC/CHO-K1 cells were plated and incubated at 37°C for 20 h prior to the addition of PD-1 effector cells and increasing concentrations of **(E)** PD-L1-blocking antibody, **(F)** SPE, or **(G)** SPE-EA. Bar graph (mean ± standard error of the mean) statistics were determined with the data of three experiments, using one-way ANOVA with Tukey’s post-hoc test; ***P < 0.001, **P < 0.01, *P < 0.05, n.s, not significant, compared with the control.

To evaluate the selective PD-1/PD-L1 blockade properties of SPE, we performed competitive PD-1/PD-L1 ELISA-binding assays. As a positive control, PD-L1-blocking antibody was used, which recognizes the extracellular domain of hPD-L1 and blocks the binding of PD-L1 to PD-1 (25). SPE blocked the binding of PD-L1 to PD-1 in a concentration-dependent manner (**Figures 1A, B**). SPE 50 μg/mL showed similar inhibitory effect compared to that of PD-L1-blocking antibody 10 μg/mL. Additionally, we tested the blocking effects of SPE on the other immune checkpoint CTLA-4/CD80 by competitive ELISA. The results confirmed that SPE cannot block the CTLA-4/CD80 interaction (**Supplementary Figure S1**). This result suggests that SPE specifically blocks the PD-1/PD-L1 interaction. Next, we also investigated the ability of the methylene chloride (MC), ethyl acetate (EA), and n-butanol (BuOH) fractions of SPE to inhibit the PD-1/PD-L1 interaction. Among these, SPE-EA showed the strongest PD-1/PD-L1 blockade, which was much higher than that for the other fractions (**Figure 1C**). These findings confirmed that SPE (50 µg/mL) and SPE-EA (50 µg/mL) inhibited the PD-1/PD-L1 interaction at rates of 41.93 and 62.89%, respectively (**Figures 1B, C**).

### Cytotoxicity Effects of SPE

Previous studies have revealed that the PD-1/PD-L1 axis inhibits T-cell function and recovery of T-cell dysfunction is important for enhancing immune function against cancer (2, 3, 6). Therefore, in subsequent assays, T-cell function was elucidated in co-cultured cell model systems using CHO-K1 cells engineered to stably express hPD-L1 and TCR agonist (aAPC/CHO-K1 cells) and Jurkat cells (T-lymphocyte-like cell line) engineered to stably express hPD-1 and TCR-inducible NFAT-luciferase reporter construct (PD-1 effector cells). Initially, to define the maximum acceptable concentration of SPE for use in cell-based assays, the toxicity of SPE was assessed using the CCK assay. Co-cultured cells were exposed to increasing SPE concentrations (0–400 μg/mL) for 24 h. It was found that SPE was not cytotoxic to aAPC/CHO-K1 or PD-1 effector cells at concentrations below 50 μg/mL. However, at a concentration of 100 μg/mL, SPE showed cytotoxicity (**Figure 1D**). Thus, subsequent experiments were conducted with SPE concentrations less than 50 μg/mL.

### SPE and SPE-EA Blockade of the PD-1/PD-L1 Immune Checkpoint Pathway and T-Cell Function

It has been well-known that the NFAT family of transcription factors play important roles in T-cell activation. To elucidate whether the blocking of on PD-1/PD-L1 axis by SPE and SPE-EA may affect T-cell functional activity, their effects on NFAT-mediated luciferase activity we reevaluated using a PD-1/PD-L1 blockade bioassay. Co-culture of aAPC/CHO-K1 cells and PD-1 effector cells induced PD-1/PD-L1 interaction in cell model systems; subsequently, TCR signaling and NFAT-mediated luciferase activity were found to be suppressed by PD-1. Agents that can efficiently interfere with the PD-1/PD-L1 interaction will activate the expression of reporter genes and thereby increase luciferase activity. We confirmed that PD-L1-blocking antibody improved TCR signaling, with an EC_50_ value of 0.306 μg/mL (**Figure 1E**). Both SPE and SPE-EA dose-dependently induced the activity of luciferase, demonstrating an antagonistic effect on the PD-1/PD-L1 immune checkpoint. Additionally, SPE and SPE-EA improved TCR signaling, with EC_50_ values of 27.2 μg/mL and 1.08 μg/mL, respectively (**Figures 1F, G**).

### SPE Blockade of the PD-1/PD-L1 Immune Checkpoint Pathway and Enhancement of T-Cell-Mediated Killing of Colorectal Cancer Cells

The PD-1/PD-L1 immune checkpoint pathway was previously reported to suppress T-cell activity and function, allowing immune evasion of cancer cells (33). As shown in **Figure 1**, we confirmed that SPE could block the PD-1/PD-L1 immune checkpoint pathway and improve T-cell activation, thus, we hypothesized that SPE would induce killing of cancer cells mediated by T-cell activation.

**Figure 2:**
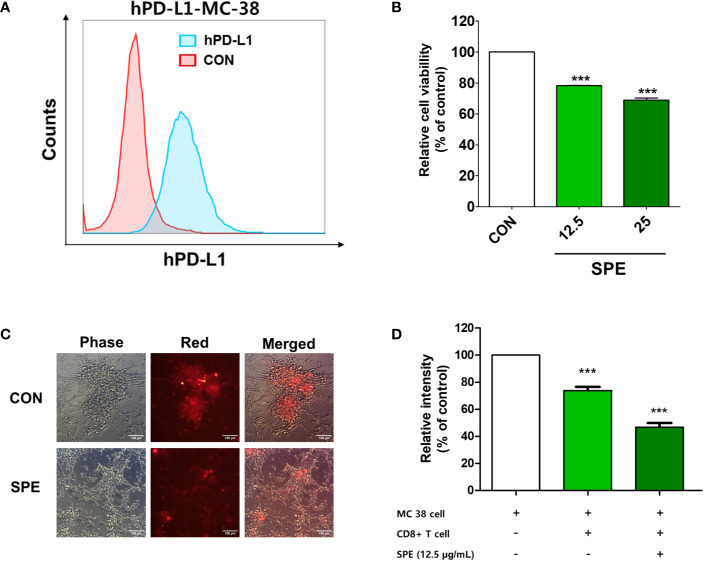
Cytotoxic and T-cell-mediated killing effect of SPE on colorectal cancer cells. **(A)** PD-L1 expression on the surface of humanized PD-L1-expressing MC38 cells (hPDL1-MCs) was determined using flow cytometry. **(B–D)** To measure the immunological activity of SPE, hPDL1-MCs (target cells) were co-cultured with humanized PD-1 mouse splenocytes **(B, C)** and CD8+ T-cells from the tumor **(D)** (hPD1-MSs; effector cells) at an effector cell-to-target cell ratio of 5:1 and were treated with SPE or 50% DMSO (control). **(B)** After 72 h of incubation, surviving hPD1-MSs in a 96-well plate underwent the CCK assay to assess cell viability. **(C)** Co-cultured CellTrace™ Far Red-labeled hPDL1-MCs were treated with SPE (25 μg/mL) or 50% DMSO (control), and the findings were compared with those in the absence of effector cells. The images were obtained under a fluorescence microscope. **(D)** The cytotoxicity of CD8+ T-cells against hPD-L1-MCs at an effector cell-to-target cell ratio of 5:1 was analyzed using an LDH assay kit (percentages calculated according to kit instruction). ***P < 0.001, compared with the control.

To measure the immunological activity of SPE, we used humanized PD-1 mouse splenocytes (hPD1-MSs) as effector cells and humanized PD-L1-expressing MC38 cells (hPDL1-MCs) as target cells. PD-L1 expression on the surface of hPDL1-MCs was determined using flow cytometry (**Figure 2A**). SPE was not cytotoxic to hPDL1-MCs at concentrations 25 μg/mL (**Supplementary Figure S2**). Thus, subsequent experiments were conducted with SPE concentrations 25 and below12.5 μg/mL.

Cultured hPDL1-MCs were labeled with CellTrace™ Far Red and were then co-cultured with hPD1-MSs and treated with SPE. At an effector cell-to-target cell ratio of 5:1, SPE treatment effectively reduced the proliferation of hPDL1-MCs (**Figures 2B, C**). In addition, co-cultivation of CD8+ T-cells isolated from the tumor with hPDL1-MCs indicated that SPE treatment induces the death of cancer cells by CD8+ T-cell activation (**Figure 2D**). These results suggest that SPE increased T-cell activity and function by blocking the PD-1/PD-L1 immune checkpoint pathway.

### PD-1/PD-L1 Interaction Blockade by Seven Components From SPE

**Figure 3:**
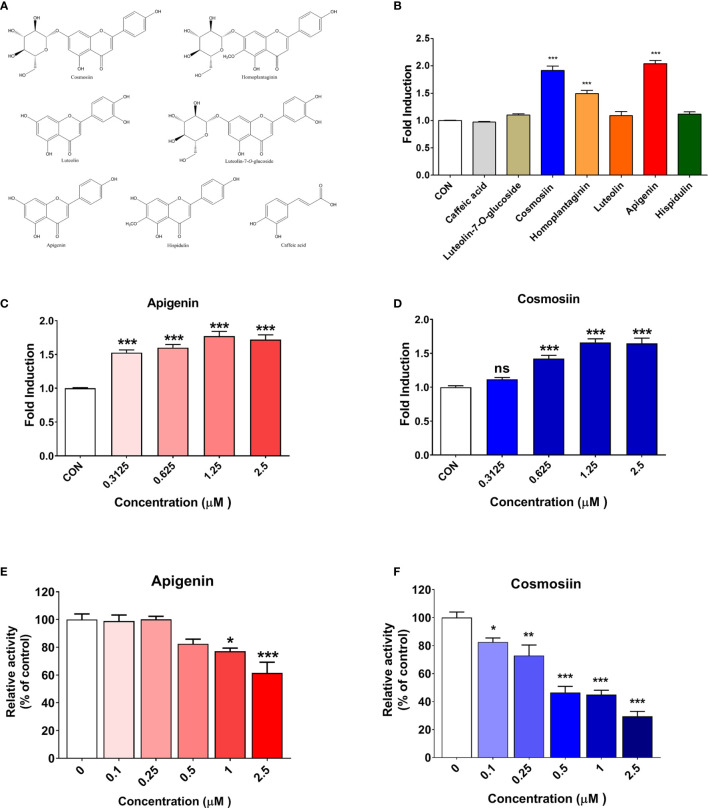
PD-1/PD-L1 interaction blockade and T-cell activation enhancement by SPE components *in vitro*. **(A)** Chemical components of SPE. **(B)** The ability of seven components from SPE to enhance T-cell functional activity was assessed using a PD-1/PD-L1 blockade bioassay. These seven components were used at a concentration of 2 µM. **(C, D)** A PD-1/PD-L1 blockade bioassay was performed to measure the inhibitory activity of apigenin and cosmosiin. PD-L1 aAPC/CHO-K1 cells were plated and incubated at 37°C for 20 h prior to the addition of PD-1 effector cells and increasing concentrations of apigenin or cosmosiin. **(E, F)** Competitive ELISA was performed using a PD-1/PD-L1 inhibitor ELISA-binding assay after treatment with the indicated concentrations of **(E)** apigenin and **(F)** cosmosiin. Bar graph (mean ± standard error of the mean) statistics were determined with data of three experiments, using one-way ANOVA with Tukey’s post-hoc test; ***P < 0.001, **P < 0.01, *P < 0.05, n.s, not significant, compared with the control.

The ability of seven SPE components, which were selected based on the previous reports (21, 27, 34–36), to enhance T-cell functional activity was assessed using a PD-1/PD-L1 blockade bioassay. These seven components (**Figure 3A**) were used at a concentration of 2 µM, and among them, apigenin and cosmosiin showed the strongest PD-1/PD-L1 blockade with enhanced T-cell functional activity, which was much higher than that for the other SPE components (**Figure 3B**). Treatment with apigenin and cosmosiin increased T-cell functional activity by 2.03-fold and 1.91-fold, respectively, when compared with the findings in controls (**Figure 3B**). Furthermore, the dose-dependent increase in T-cell functional activity was evaluated using a PD-1/PD-L1 blockade bioassay for apigenin (**Figure 3C**) and cosmosiin (**Figure 3D**), and both apigenin and cosmosiin increased T-cell functional activity in a dose-dependent manner. We further investigated PD-1/PD-L1 interaction blockade by apigenin and cosmosiin, and we confirmed PD-1/PD-L1 interaction blockade using a PD-1/PD-L1 inhibitor ELISA-binding assay. Moreover, the dose-dependent inhibition of the PD-1/PD-L1 interaction was assessed for apigenin (**Figure 3E**) and cosmosiin (**Figure 3F**). Among the components, cosmosiin showed the strongest PD-1/PD-L1 blockade.

On assessing the structure–activity relationships of seven flavonoids, we found that the activities of apigenin, cosmosiin, homoplantaginin, and hispidulin were higher than the activities of the other flavonoids, because of the absence of substitutes at C-3’ of the skeleton. Therefore, non-substitution at C-3’ of the skeleton was considered an important functional aspect. Moreover, we compared apigenin/cosmosiin and homoplantaginin/hispidulin and found that the activities of apigenin/cosmosiin were higher than the activities of homoplantaginin/hispidulin. Therefore, non-substitution at C-6 was considered another important functional aspect. Interestingly, the activities of cosmosiin and homoplantaginin were higher than the activities of their skeletons (apigenin and hispidulin). Therefore, a monosaccharide group at C-7 was considered to play an important role in enhancing activity. Overall, among SPE components, apigenin, and cosmosiin were the most effective for blocking the PD-1/PD-L1 interaction. We tested the blocking effects of cosmosiin on the other Immune checkpoint CTLA-4/CD80 by competitive ELISA. The results confirmed that cosmosiin cannot block the CTLA-4/CD80 interaction (**Supplementary Figure S1**). This result suggests that cosmosiin specifically blocks the PD-1/PD-L1 interaction.

### Chemical Composition of SPE on High-Performance Liquid Chromatography Analysis

High-performance liquid chromatography (HPLC) analysis was performed to investigate the amounts of the four effective components/flavonoids (cosmosiin, homoplantaginin, apigenin, and hispidulin) in SPE and SPE fractions (MC, EA, and BuOH) by comparing the retention times with the findings for standard mixtures. HPLC analysis confirmed the presence of cosmosiin (t^R^ = 18.514 min; peak 1), homoplantaginin (t^R^ = 19.818 min; peak 2), apigenin (t^R^ = 34.962 min; peak 3), and hispidulin (t^R^ = 35.885 min; peak 4) (**Supplementary Figure S3** and **Table S1**). The HPLC results indicated that hispidulin and its glycoside homoplantaginin were the primary components in SPE (12.8 mg/g and 22.0 mg/g, respectively), and subsequent extraction with EA increased the amounts of all the selected components. However, only the two glycosides cosmosiin and homoplantaginin were noted in SPE-BuOH. The amount of apigenin increased 18.3-fold in SPE-EA and that of its glycoside cosmosiin increased 7.8 fold and 5.8 fold in SPE-EA and SPE-BuOH, respectively, when compared with the amounts in SPE.

### The Affinity of Small Molecule Cosmosiin With PD-1 and PD-L1

**Figure 4:**
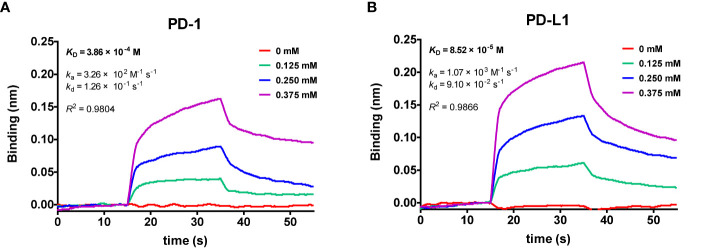
Global kinetic analysis of cosmosiin binding to biotinylated **(A)** PD-1 and **(B)** PD-L1 immobilized on a streptavidin biolayer interferometry (BLI) sensor. The kinetics of cosmosiin for immobilized PD-1 and PD-L1 were monitored with increasing concentration of cosmosiin (0, 0.125, 0.25, and 0.375 mM), dissolved in PBS buffer (pH 7.3) containing 1% DMSO.

The affinity of cosmosiin with PD-1 and PD-L1 were evaluated by kinetic model using BLItz system from Pall ForteBio (Menlo Park, USA). As shown in **Figure 4**, the increase of cosmosiin concentration led to a wavelength shift in the BLItz sensorgram, indicating an increase in the cosmosiin interacting with the biotinylated PD-1 and PD-L1, respectively, immobilized on the streptavidin sensor. The equilibrium dissociation constants (K_D_) of cosmosiin to PD-1 and PD-L1 were 386 and 85 μM with co efficient of determinations, *R^2^*, of 0.9804 and 0.9866, respectively, showing high goodness of fit to 1:1 binding model and that cosmosiin has 4.5-fold higher affinity to PD-L1 than PD-1.Furthermore, the k_a_ and k_d_ values of cosmosiin to PD-1 and PD-L1 in **Figure 4** indicate that the higher affinity of cosmosiin to PD-L1 results from its higher binding rate to PD-L1 rather than insignificant difference in dissociation rates.

### Protein–Ligand Docking Simulation and Pharmacophore Analysis of SPE Components

**Figure 5:**
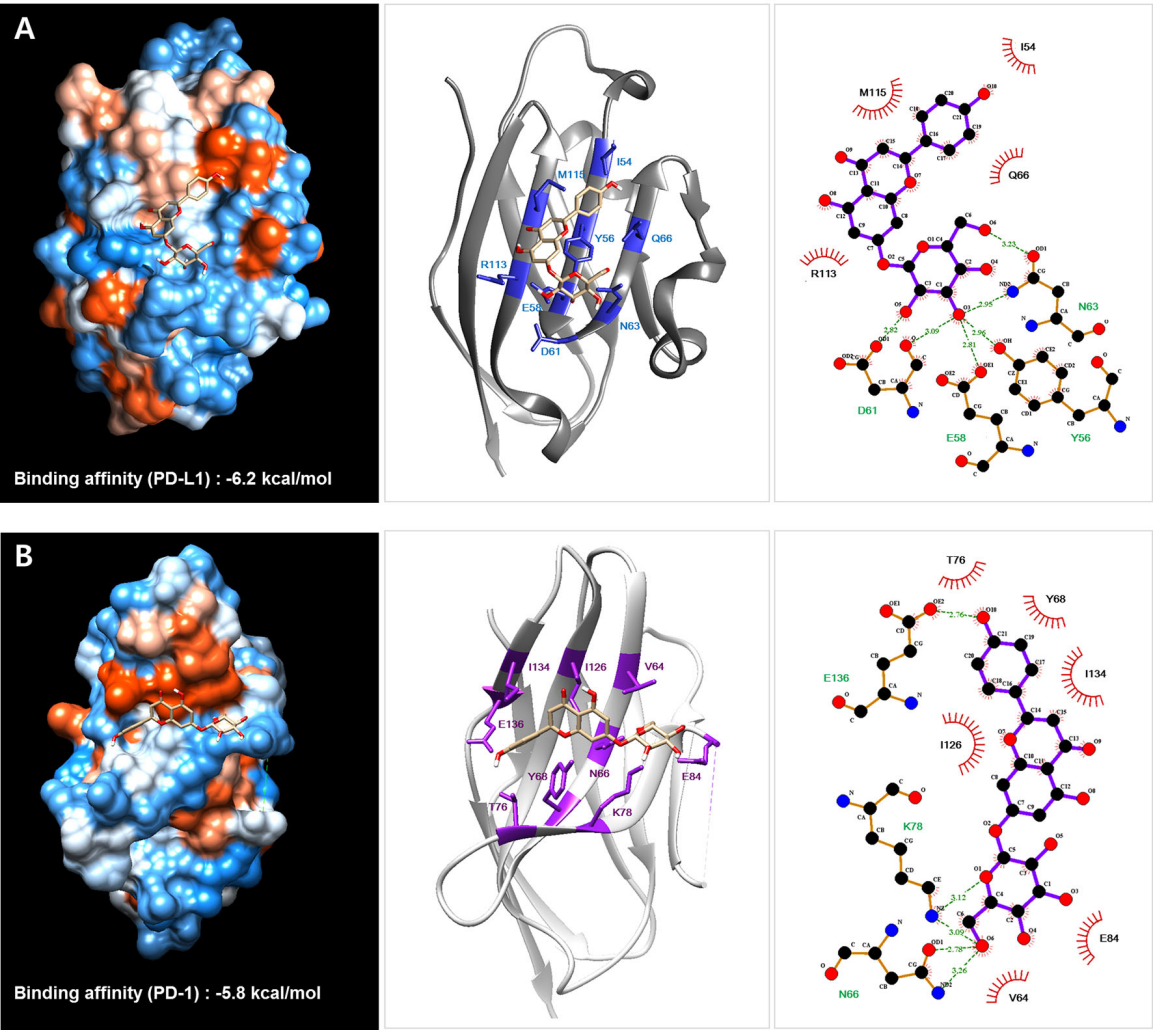
Protein–ligand docking simulation between PD-L1 **(A)**/PD-1 **(B)** and the *Salvia plebeia* R. Br. ethanol extract (SPE) component cosmosiin. Binding models were obtained through docking simulation of cosmosiin on PD-L1 and PD-1 derived from the PD-1/PD-L1 complex (PDB code 4ZQK) using AutoDock Vina. The hydrogen bonds and hydrophobic interactions between PD-L1/PD-1 and cosmosiin were analyzed using LigPlot+.

Previous studies have reported a crystal structure showing the interaction between hPD-1 and hPD-L1 (PDB code: 4ZQK) (28) and the binding region of small molecules in PD-1 and PD-L1 (37). According to this information, we investigated the binding affinity of cosmosiin, which was found to be effective in PD-1/PD-L1 interaction blockade, to PD-L1 by using protein–ligand docking simulation with AutoDock Vina (**Figure 5**). The predicted binding affinities of cosmosiin to PD-L1 and PD-1 were −6.2 and −5.8 kcal/mol, respectively, indicating that cosmosiin binds to PD-L1 more strongly than PD-1. We further investigated the interactions between cosmosiin and amino acid residues in the PD-L1-binding pocket to assess PD-1/PD-L1 interaction blockade by cosmosiin, using pharmacophore analysis with LigPlot+ software. It was found that four amino acid residues (Y56, E58, D61, and N63) in PD-L1 interact with the glucoside of cosmosiin through hydrogen bonds and another four amino acid residues (I54, Q66, M115, and R113) form hydrophobic interactions with the backbone of cosmosiin (apigenin). This pharmacophore analysis revealed that the backbone of cosmosiin interacts with a hydrophobic pocket on the PD-L1 surface (consisting of Y56, E58, R113, M115, and Y123), which has been identified in a previous study (28).

### Anti-Tumor Effect of SPE in hPD-L1 Knock-In MC38 Tumor-Bearing Humanized PD-1 Mouse Model

**Figure 6:**
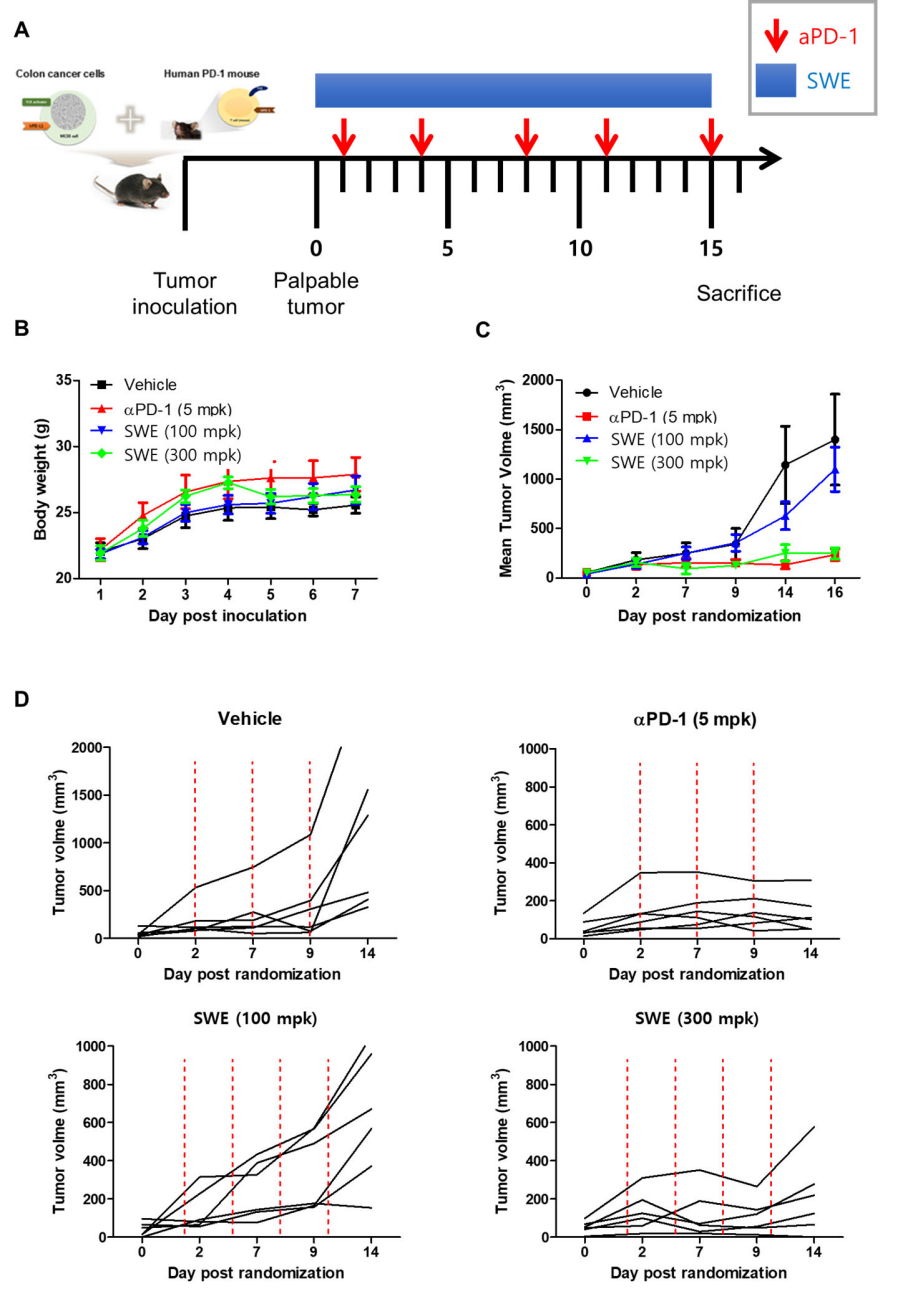
SPE attenuated growth of hPD-L1 MC38 tumors in hPD-1 knock-in C57BL/6 mice. **(A)** Experimental scheme. The figure was adapted from a previous study and slightly modified (38). **(B)** Mean bodyweight. **(C)** Mean tumor growth curves. **(D)** The individual tumor growth over time for plot. MC38 cells expressing hPD-L1 (1.5 x 10^5^ cells/mouse) were inoculated subcutaneously (day -14) into hPD-1 knock-in C57BL6/J mice. Once tumors became palpable (~100 mm^3^), tumor-bearing mice were randomized into groups of 6 animals per group: an untreated control group (no tumor cells injected-group), and groups injected with MC38 tumor cells plus vehicle (PBS, 10 mL/kg, q.d., i.g.), Anti-PD-1 antibody with pembrolizumab (5 mg/kg, b.i.w.x2, i.p.), a low or high dose of SPE (100 or 300 mg/kg, q.d., i.g.). On days 1, 4, 8, 11, and 15 post implantation, mice were dosed intraperitoneally (i.p.) with PBS or anti-PD-1 antibody. Intragastric (i.g.) injection were daily given with SPE for 15 days. On day 16 post administration, all mice were sacrificed for *in vivo* analysis. Statistics were analyzed using two-way ANOVA with Bonferroni post-hoc test; ***P < 0.001, compared with the vehicle group.

**Figure 7:**
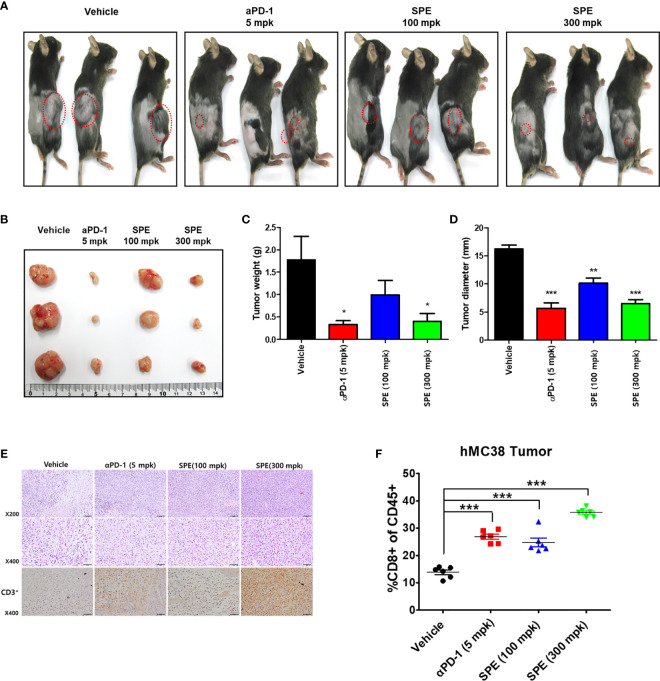
Anti-tumor efficacy of SPE on hPD-L1 MC38 tumor growth in hPD-1 knock-in mice. **(A)** Representative images show the tumor development at 16 days after treatment. Mice were inoculated s.c. with hPD-L1 MC38 cells and were administrated with aPD-1 (5 mg/kg, i.p.) or SPE (100, 300 mg/kg, i.g.) (n=3). Tumor mice received an equivalent volume of vehicle (200 µL). **(B)** Image of subcutaneous tumor mass on day 16 (scale bars, 1 cm) (n=3). **(C)** Mean tumor weight (n=3). **(D)** Mean tumor diameter (n=3). **(E)** Immunohistochemistry staining of CD3+ in humanized PD-1 mouse model of tumor. **(F)** FACS analysis of tumor CD8+ T-cell populations from human PD-L1 knock in MC 38 tumor-bearing model of humanized PD-1 mice. Percentages of polyclonal CD8+ T-cells of CD45+ cells in tumor. Bar graph (mean ± standard error of the mean) statistics were determined with one-way ANOVA with Tukey’s post-hoc test; ***P < 0.001, **P < 0.01, *P < 0.05, compared with the vehicle group.

*In vivo* antitumor efficacy of SPE was evaluated on the hPD-L1 knock-in MC38 tumor-bearing humanized PD-1 mouse model. During the SPE and anti-hPD-1 antibody treatment period, tumor volumes were measured twice a week and initiation of therapy the tumor volume reached 100 mm^3^ (day 14), mice were randomized into groups of six animals per group (**Figure 6A**). Animals were treated with positive control, as anti-hPD-1 antibody (5 mg/kg) was intraperitoneal (i.p.) injection twice a week. During the experiment, SPE treatment did not reduce body weight, and there were no symptoms of toxicity (**Figure 6B**). SPE (100 and 300 mg/kg) was treated intragastric administration using disposable feeding needle each day (**Figure 6A**). SPE at a dose of 100 and 300 mg/kg showed a mean tumor growth inhibition with inhibition rates of 44.9 and 77.8% on day 16 (**Figure 6C**). The positive control anti-hPD-1 antibody showed a mean tumor growth inhibition rates was 88.3% on day 16. We found that SPE did show a dose dependent inhibition of tumor growth (**Figures 7A–D**).

Recent studies have reported that several drugs in the tumor microenvironment can enhance T-cell infiltration and activation (39, 40). To determine whether SPE enhanced T-cell cytotoxic activity, we further examined the tumor infiltrating lymphocytes (TILs) and relative activation in tumor tissues derived from humanized PD-1 mouse model. Compared with the control groups, we observed that SPE increased the number of CD3+ TILs (**Figure 7E**). Overall, these results demonstrate that SPE treatment improves the number of TILs with anti-tumor immune activity. In addition, anti-PD-1 reported that PD-1/PD-L1 blockade promotes CD8+ T-cell infiltration. To characterize changes in the tumor microenvironment associated with the antitumor activity of SPE and anti-PD1, we performed the phenotype of immune cells as CD8+ T-cells in a tumor microenvironment by flow cytometry to address whether SPE has reached this point. Flow cytometry analysis for CD8 + confirmed that it showed an increase in infiltration of CD8+ T-cells in the group treated with SPE and anti-PD1. As a result, the CD8+ T-cell infiltration in the SPE (300 mpk) group was significantly higher than vehicle groups treatment (**Figure 7F**). This result suggests that SPE showed anti-tumor activity in hPD-L1 knock in MC 38 tumor-bearing humanized PD-1 mouse model.

## Discussion

Immune checkpoint blockade by anti-PD-1 or anti-PD-L1 antibodies has been shown to be a promising therapeutic approach for human cancer (3, 4). Pembrolizumab, nivolumab, atezolizumab, avelumab, and durvalumab have been approved by the FDA for cancer immunotherapy, and they are being currently used in clinical practice (7). However, the use of immune checkpoint blockade antibodies has many issues (8), and only few patients respond to these agents. Thus, there is a need to identify other approaches to complement the current approach. A promising alternative to antibody-based immunotherapy is the use of low-molecular-weight inhibitors, which might become an affordable cancer therapy in the future. Small molecules capable of overcoming the above-mentioned issues can become alternative or complementary therapies for cancer. Many patents of small molecules considered to be active against PD-1/PD-L1 have been filed (10, 41–43). To date, among all the disclosed small molecules, only one small molecule (CA-170) has been assessed in clinical trials (42, 43). However, no small molecules have been approved by the FDA as inhibitors of the PD-1/PD-L1 pathway.

We assessed the ability of SPE to block the PD-1/PD-L1 pathway using ELISA-binding assays and found that SPE and SPE-EA blocked the PD-1/PD-L1 pathway in a concentration-dependent manner. In addition, we evaluated the ability of SPE and SPE-EA to enhance T-cell functional activity using PD-1/PD-L1 blockade bioassays and confirmed that SPE and SPE-EA induced the activity of luciferase, indicating an antagonistic effect towards the PD-1/PD-L1 immune checkpoint pathway, and improved TCR signaling. We further assessed the ability of seven components from SPE to enhance T-cell functional activity using PD-1/PD-L1 blockade bioassays and confirmed that apigenin and cosmosiin showed the strongest PD-1/PD-L1 blockade with enhanced T-cell functional activity, which was much higher than that for the other SPE components.

We further identified the constituents of SPE using HPLC and confirmed that apigenin and cosmosiin blocked the PD-1/PD-L1 pathway, using ELISA-binding assays and cell-based blockade bioassays. In addition, we investigated the amounts of the identified phytochemicals and found that their amounts ranged from 0.3 to 22.0 mg/g in SPE and that the active components apigenin and cosmosiin were relatively abundant 0.3 mg/g and 1.2 mg/g, respectively. Moreover, the amounts of the identified phytochemicals ranged from 5.5 to 123 mg/g in SPE-EA and the active components apigenin and cosmosiin were highly abundant at 5.5 and 9.4 mg/g, respectively. Furthermore, we confirmed that SPE-EA showed the strongest PD-1/PD-L1 blockade with enhanced T-cell functional activity, which was much higher than that for SPE. This finding might be related to the higher amounts of apigenin and cosmosiin, which strongly blocked PD-1/PD-L1, in SPE-EA than in SPE. We additionally assessed the structure–activity relationships of the flavonoids and found that non-substitution at C-3’/C-6 of the skeleton was an important functional aspect and that a monosaccharide group at C-7 could enhance activity. These findings may be useful for the evaluation of the structure–activity relationships of other flavonoids.

We also investigated the binding of SPE component cosmosiin to PD-1 and PD-L1 by biolayer interferometry (BLI), concluding that cosmosiin binds to PD-L1 more strongly than PD-1. Protein–ligand docking simulation supported the BLI result by exhibiting a lower binding score for PD-L1. The pharmacophore analysis identified four hydrophobic interactions (I54, Q66, R113, and M115) and four hydrogen bonds (Y56, E58, D61, and N63) between PD-L1 and cosmosiin, suggesting that cosmosiin blocks a hydrophobic pocket in PD-L1 surrounded by Y56, E58, R113, M115, and Y123, which is one of three hot spots on the PD-L1 surface that interact with PD-1 (28). Based on these results, the observed biological activities of SPE are considered because of the existence of a cosmosiin. Previously, cosmosiin has been reported to possess anticancer activity in various cancer cells, such as gastric cancer (44), melanoma (45), and human leukemia, through apoptotic and anti-proliferative regulation. To our knowledge, this is the first study to report the ability of SPE and its component cosmosiin to inhibit PD-1/PD-L1interaction. However, there is limitation on the PD-1/PD-L1-blocking effect of cosmosiin. Based on our observation, cosmosiin exhibited relatively high K_D_ value (>85 μM) representing the low binding affinity of cosmosiin for PD-L1. Given the widely studied nature of these compounds for several conditions including cancer, the specificity of cosmosiin and other ingredients in SPE to only PD-L1 could be difficult to establish with the native compounds. Therefore, chemical modifications of the parent compounds for drug development would be required to overcome this limitation and it is necessary to experimentally prove our hypothesis using various ligand binding assays.

Although chemical modification studies are needed to clarify the pharmacological mechanisms of SPE’s components, particularly cosmosiin, the present study confirmed that cosmosiin has potent immunotherapeutic properties against cancer *via* inhibiting PD-1/PD-L1 pathway.

Efforts have been undertaken for the development of therapeutic antibodies targeting hPD-1/hPD-L1 to treat various types of human cancer. However, until now, there is no animal model suitable for evaluating the antitumor efficacy of these antibodies targeting hPD-1/hPD-L1 (24). Thus, in this study investigated anti-tumor effect of SPE, using hPD-L1 knock-in MC38 tumor-bearing humanized PD-1 mouse model to study successfully established tumor immunotherapies. We found that SPE showed dose-dependent inhibition of mean tumor growth (**Figures 6C**). As shown in **Figure 6D**, a treatment with low dose of SPE (100 mg/kg, lower left graph) seems to exhibit mild inhibitory effect on the tumor growth after 9 days randomization, however, high dose of SPE (300 mg/kg, lower right graph) showed the suppressive effect similar to anti-PD-1-treated group (5 mpk). In addition, the present inventors confirmed that tumor weight of SPE treated groups was significantly reduced compared to the vehicle group (**Figure 7**).

To characterize changes in the tumor microenvironment associated with the anti-tumor activity of SPE and anti-PD1, we immunized as CD8+ T-cells in the tumor microenvironment by flow cytometry to determine whether SPE has reached this point. Flow cytometry analysis for CD8+ showed increased infiltration of CD8+ T-cells in the group treated with SPE and anti-PD1. Consequently, CD8+ T-cell infiltration in the SPE group was significantly higher than vehicle group treatment (**Figure 7**). These results suggest that SPE showed ant-tumor activity in hPD-L1 knock-in MC38 tumor-bearing humanized PD-1 mouse model. Thus, SPE appears to provide a promising cancer immunotherapeutic. The present *in vitro* and *in vivo* assay using humanized PD-1 mouse model include preclinical strategies for the identification and development of small molecule-based anticancer drugs targeting the PD-1/PD-L1 immune checkpoint pathway.

## Conclusions

We showed the anticancer effects of SPE and its components, especially cosmosiin. SPE blocks the PD-1/PD-L1 interaction in a concentration-dependent manner and enhances T-cell-mediated anti-tumor activity. Additionally, cosmosiin strongly blocks the PD-1/PD-L1 interaction. Our data highlight the use of hPD-L1 knock-in MC38 tumor-bearing humanized PD-1 mouse model to comprehensively study the antitumor effects of SPE. In addition, to our knowledge, this is the first time the effect of SPE was studied with immunotherapy for targeting PD-1/PD-L1 interaction. The results of this study could help in the development of potent anticancer drugs that target the PD-1/PD-L1 immune checkpoint pathway.

## Data Availability Statement

The raw data supporting the conclusions of this article will be made available by the authors, without undue reservation.

## Ethics Statement

The animal study was reviewed and approved by the Institutional Animal Care and Use Committee (IACUC) of the Korea Institute of Oriental Medicine (KIOM). Written informed consent was obtained from the owners for the participation of their animals in this study.

## Author Contributions

J-GC and YK designed the study, conducted the experiments, and wrote the manuscript. JK, TK, WL, TO, CJ, and SK performed the experiments. H-SC supervised the research and wrote the manuscript. All authors contributed to the article and approved the submitted version.

## Funding

This research was supported by Grant number KSN2013230 from the KIOM (Korea Institute of Oriental Medicine), provided by the Ministry of Science and ICT, Republic of Korea.

## Conflict of Interest

The authors declare that the research was conducted in the absence of any commercial or financial relationships that could be construed as a potential conflict of interest.
