# Supplementary material for: Anticancer Effect of Salvia plebeia and Its Active Compound by Improving T-Cell Activity via Blockade of PD-1/PD-L1 Interaction in Humanized PD-1 Mouse Model
Source: Front Immunol. 2020 Nov 5;11:598556. doi: 10.3389/fimmu.2020.598556 (PMC7674495; doi:10.3389/fimmu.2020.598556)
Supplement: Supplementary file 1 [file Table_1.docx]

Supplementary Material


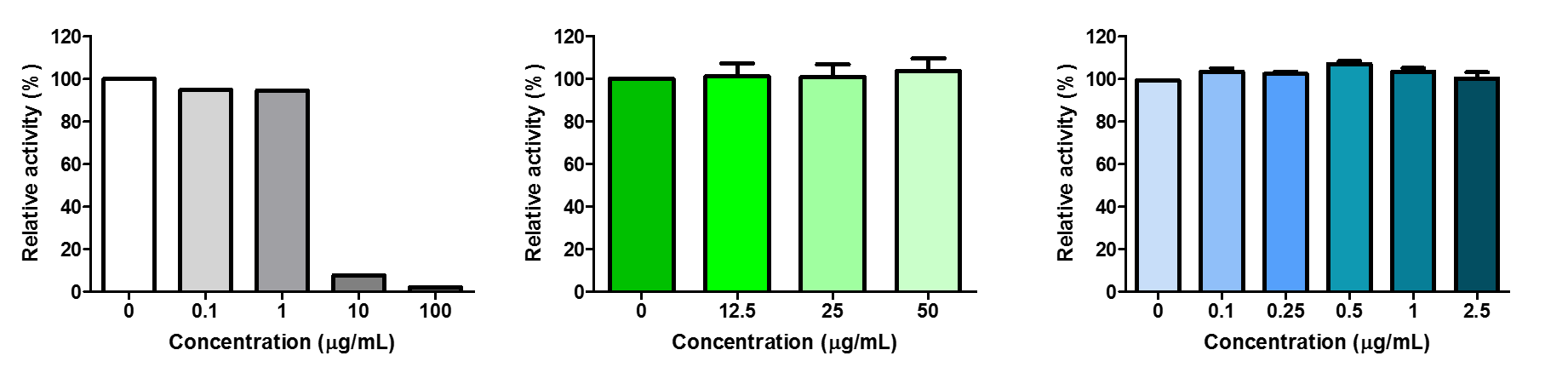


**Figure S1.** Competitive ELISA was performed using a CTLA-4/CD80 inhibitor ELISA-binding assay after treatment with the indicated SPE and cosmosiin.


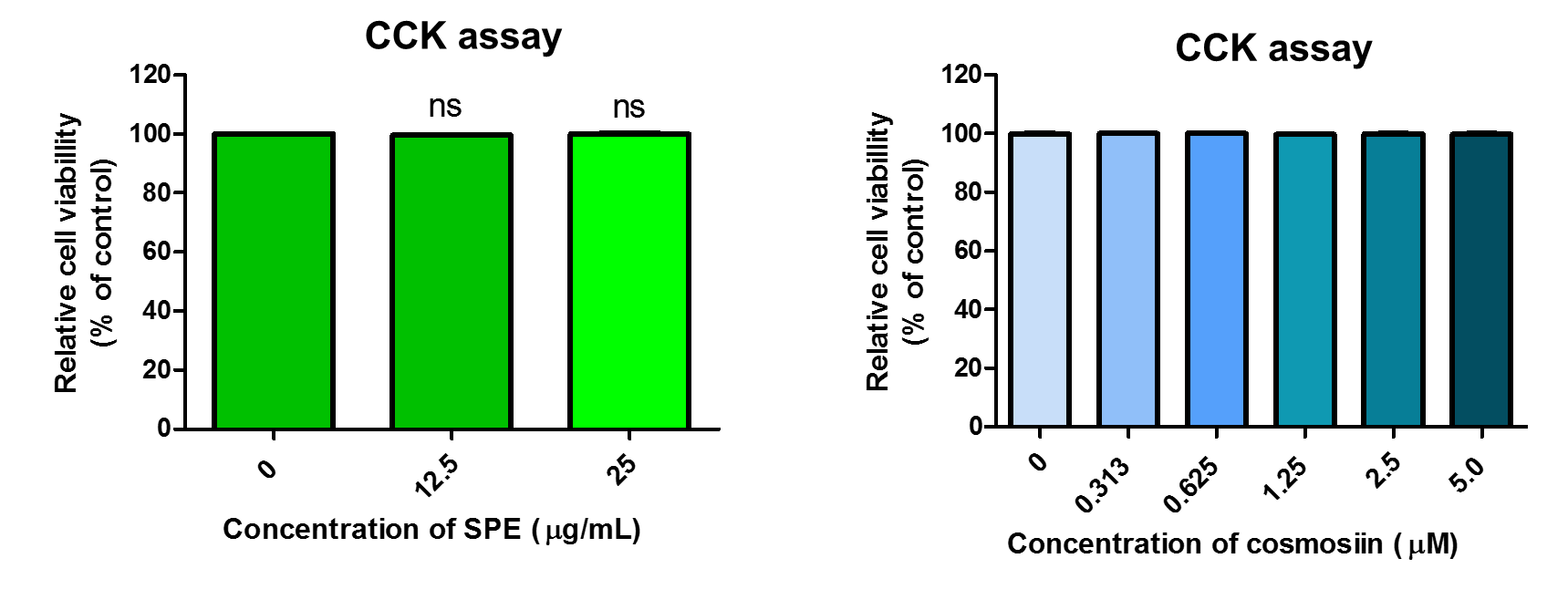


**Figure S2.** The viabilities of hPDL1-MCs cells were assessed using the Cell Counting Kit-8 (CCK) assay after treatment with the indicated concentrations of SPE and cosmosiin for 24 h.


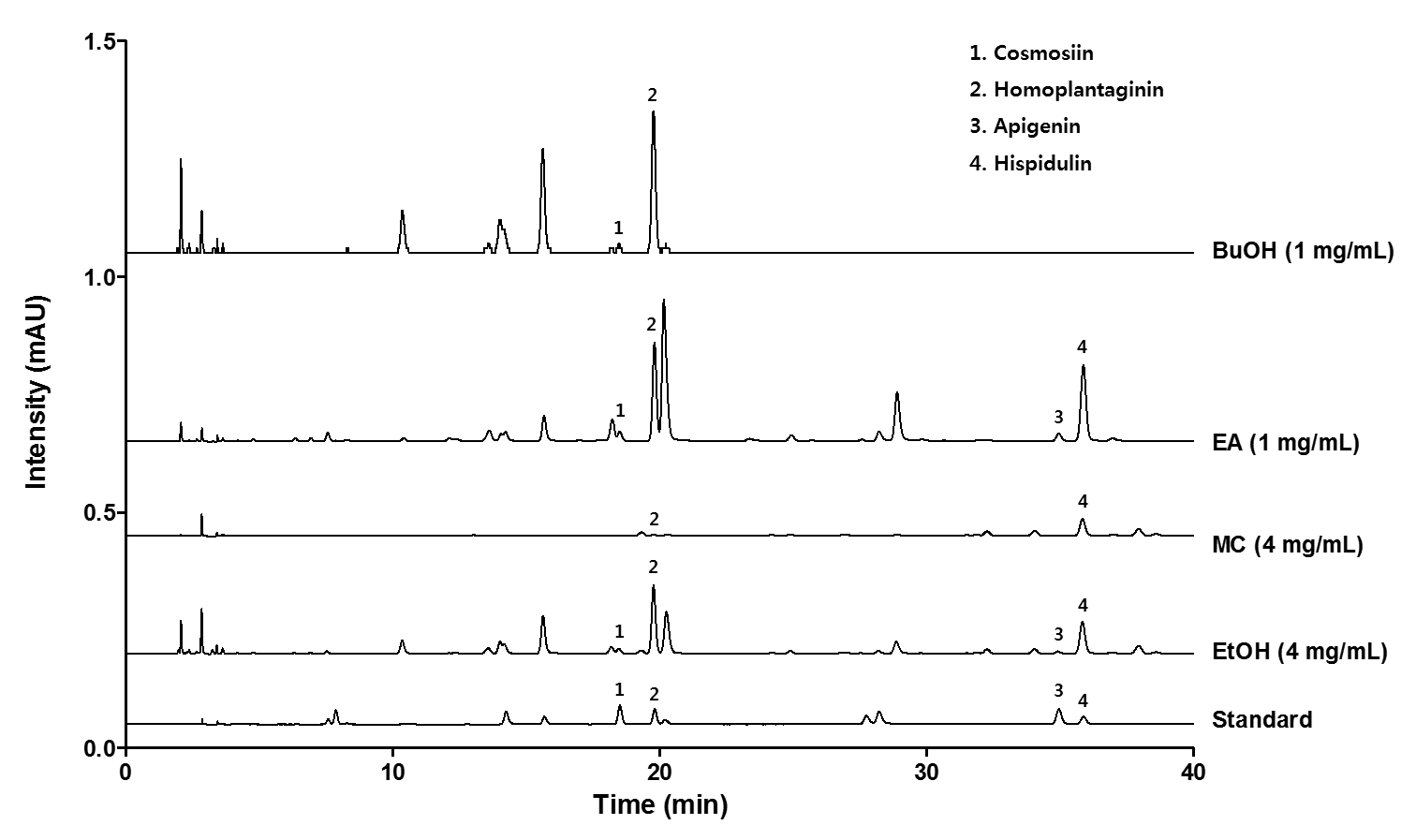


**Figure S3.** High-performance liquid chromatography profiles of cosmosiin, homoplantaginin, apigenin, and hispidulin from *Salvia plebeia* R. Br. ethanol extract (SPE) and its methylene chloride (MC), ethyl acetate (EA), and n-butanol (BuOH) fractions monitored at 265 nm. Standard cosmosiin, homoplantaginin, apigenin, and hispidulin were used for reference.

**Table S1.** The amounts of cosmosiin, homoplantaginin, apigenin, and hispidulin in *Salvia plebeia* R. Br. ethanol extract and its fractions.

| **No.** | **Component** | **Retention**  **Time (min)** | **Amount**  **(mg/g extract)** | | | |
| --- | --- | --- | --- | --- | --- | --- |
|  |  |  | **Ethanol** | **Methylene chloride** | **Ethyl acetate** | **Butanol** |
| **1** | **Cosmosiin** | 18.514 | 1.2 | - | 9.4 | 7.0 |
| 2 | Homoplantaginin | 19.818 | 22.0 | 0.4 | 123 | 180 |
| 3 | Apigenin | 34.962 | 0.3 | - | 5.5 | - |
| 4 | Hispidulin | 35.885 | 12.8 | 6.2 | 118 | - |
